# Frequency of Subclinical Hypothyroidism in Patients With Chronic Kidney Disease

**DOI:** 10.7759/cureus.25864

**Published:** 2022-06-12

**Authors:** FNU Anum, Aasta Kumari, Mehak Gul, Shilpa Bai, Muhammad Haseeb, Kanza Mirza Maqsood, Amna Jamil, Faizan Shaukat, Maha Jahangir

**Affiliations:** 1 Internal Medicine, Peoples University of Medical and Health Sciences for Women, Nawabshah, PAK; 2 Internal Medicine, Chandka Medical College, Larkana, PAK; 3 Internal Medicine, Jinnah Sindh Medical University, Karachi, PAK; 4 Internal Medicine, Allama Iqbal Medical College, Lahore, PAK; 5 Internal Medicine, Jinnah Postgraduate Medical Centre, Karachi, PAK; 6 Internal Medicine, Dow University of Health Sciences, Karachi, PAK

**Keywords:** estimated glomerular filtration rate (egfr), thyroxine (t4), thyroid-stimulating hormone (tsh), chronic kidney disease, subclinical hypothyroidism

## Abstract

Introduction: Chronic kidney disease (CKD) is the progressive loss of function of the nephron over a long period of time. As the glomerular filtration rate falls, it leads to subclinical hypothyroidism (SCH). This cross-sectional study is carried out to measure the frequency of SCH in CKD patients in our population.

Methods: This case-control research was undertaken at the nephrology unit of the Peoples University of Medical and Health Sciences for Women in Pakistan from March 2021 to January 2022. The research included 200 volunteers with documented evidence of CKD between the ages of 18 and 60 years. A case group of 200 people without CKD was also enlisted, matched by age, gender, and comorbidities. Data were recorded in a self-structured questionnaire and analyzed using Statistical Package for the Social Sciences® software (IBM Corp., Armonk, NY).

Results: Thyroid-stimulating hormone was significantly raised in participants with CKD (4.91 ± 1.10 mIU/L vs. 3.62 ± 0.72 mIU/L; p-value < 0.0001). A significant association between SCH and CKD was established (p-value < 0.00001).

Conclusion: Due to the positive correlation between SCH and CKD, multidisciplinary management, including a team of endocrinologists and nephrologists, is advised to keep a regular check on these patients.

## Introduction

Chronic kidney disease (CKD) is the progressive loss of function of the kidney over a long period of time due to which the kidney cannot filter blood and nutrients properly. As the glomerular filtration rate (GFR) falls, it leads to an increased prevalence of subclinical hypothyroidism (SCH) [1]. Diagnosis of SCH is made on the basis of high serum thyroid-stimulating hormone (TSH) level (range: 4.2-10 µIU/ml) but normal serum free thyroxine (FT4) levels (normal value: 0.93-1.7 ng/dl) [1]. It occurs in the general population but is more prevalent in patients with CKD. An inverse relation was observed between estimated GFR and TSH levels, i.e., with progressively lower GFR, TSH was either normal or high and there was a graded increase in the probability of SCH [2].

According to an Indian population-based study, the age-adjusted incidence of end-stage renal disease is approximately 232 cases per million population per year [3]. The estimated frequency of SCH is 4-10% in the normal population, increasing to 18% in CKD patients [4]. In patients with an estimated GFR < 60 ml/min per 1.73 m^2^, there was an increased likelihood of SCH after adjusting for demographics and other associated factors [2]. Gender-wise stratification showed a higher prevalence in females as compared to their male counterparts (46.2% vs. 21.6%; p-value: 0.03) [5]. When stratified according to the duration of hemodialysis (HD), a high prevalence of SCH was seen in patients with chronic HD [5]. Higher rates of SCH have been seen in older patients as compared to patients of younger age. According to an Indian study, 74% of cases with SCH fell in the age group of 35-54 years, with an increase in the incidence observed after the age of 35 years, and 20% of cases were observed in postmenopausal females [5].

Despite the rising prevalence of CKD in our region [6], data are scarce in terms of the correlation of CKD with SCH; hence, an accurate estimation of the incidence or prevalence of CKD in local settings is understudied. This cross-sectional study is carried out to measure the frequency of SCH in CKD patients in our population.

## Materials and methods

This case-control research was undertaken at the nephrology unit of the Peoples University of Medical and Health Sciences for Women in Pakistan from March 2021 to January 2022.

Sampling technique and size

A total of 400 participants were enrolled in the study, i.e., 200 each in the case and control groups, via consecutive convenient non-probability sampling techniques.

Inclusion criteria

The case group included 200 volunteers, all of whom had documented evidence of CKD according to the staging (Table 1) and were between the ages of 18 and 60 years. A control group of 200 people without CKD was also enlisted, matched by age, gender, and comorbidities.

**Table 1:** Classification of stages of CKD CKD: chronic kidney disease; GFR: glomerular filtration rate; mL/min/1.73 m2: milliliters per minute per 1.73 square meter.

Staging of CKD	Description	GFR (mL/min/1.73 m^2^)
Stage 1	Mild kidney damage	≥90
Stage 2	Mild loss of kidney function	60-89
Stage 3a and 3b	Mild to moderate loss of kidney function	30-59
Stage 4	Severe loss of kidney function	15-29
Stage 5	End-stage renal disease	<15

Exclusion criteria

Participants who had previously used thyroid hormone-influencing medications, e.g. thyroxine, dopamine, or glucocorticoids, were excluded from the research.

Consent and ethical review board approval

Before patients were enrolled, they gave their informed consent. Before enrolling individuals, clearance was obtained from the Ethical Review Board of the Peoples University of Medical and Health Sciences for Women (PUMHSW/IRB/2021/R-04).

Data collection

Once enrolled, a self-structured questionnaire was used to note down the demographics of the participants. To compute body mass index (BMI), participants' height and weight were assessed. Blood was collected from the cubital vein of the individuals through phlebotomy after their demographics were recorded. TSH, free T4, and free triiodothyronine (free T3) were all measured in the blood. TSH levels between 5 and 10 mIU/L were characterized as SCH, with normal free T3 and FT4 levels, based on laboratory reference values.

Data analysis

The data analysis was done using Statistical Package for the Social Sciences® software, version 23.0 (IBM Corp., Armonk, NY). Age was displayed as a continuous variable with a mean and standard deviation (SD). Percentages and frequencies were used to display categorical information. An independent t-test was employed to compare TSH, T3, and T4 hormone levels. The chi-square test was used to compare the frequencies of SCH. A p-value of less than 0.05 suggested that there was a significant difference between the two groups, indicating that the null hypothesis was invalid.

## Results

The mean age of participants with and without CKD was 53.12 ± 6.24 years and 52.01 ± 7.01 years, respectively. Participants with CKD had significantly higher BMI (25.12 ± 2.51 kg/m2 vs. 22.51 ± 2.01 kg/m2; p-value < 0.0001) and creatinine levels (2.21 ± 0.8 mg/dL vs. 0.75 ± 0.25 mg/dL; p-value < 0.0001) than those without CKD. Demographics and risk factors profiles were comparable between the two groups (Table 2).

**Table 2:** Comparison of demographics and risk factor profile of participants BMI: body mass index; CKD: chronic kidney disease; DM: diabetes mellitus; HTN: hypertension; kg/m2: kilograms per square meter; mg/dL: milligrams per deciliter.

Characteristics	Participants with CKD (n = 200)	Participants without CKD (n = 200)	P-value
Age (in years)	53.12 ± 6.24	52.01 ± 7.01	0.0952
Gender			
Male	112 (56.0%)	109 (54.5%)	0.762
Smokers	51 (25.5%)	59 (29.5%)	0.370
BMI (kg/m^2^)	25.12 ± 2.51	22.51 ± 2.01	<0.0001
HTN	92 (46.0%)	89 (44.5%)	0.763
DM	84 (42.0%)	86 (43.0%)	0.839
Creatinine (mg/dL)	2.21 ± 0.18	0.75 ± 0.25	<0.0001

TSH was significantly raised in participants with CKD (4.91 ± 1.10 mIU/L vs. 3.62 ± 0.72 mIU/L; p-value < 0.0001). SCH was significantly associated with CKD (p-value < 0.00001). The thyroid profile was comparable between both groups (Table 3).

**Table 3:** Comparison of thyroid profile FT3: free triiodothyronine; FT4: free thyroxine; mIU/L: milli-international units per liter; ng/dL: nanograms per deciliter; pg/dL: picograms per deciliter; SCH: subclinical hypothyroidism; TSH: thyroid-stimulating hormone.

Thyroid hormones	Participants with CKD (n = 200)	Participants without CKD (n = 200)	P-value
TSH (mIU/L) (normal: 0.5-5.0 mIU/L)	4.91 ± 1.10	3.62 ± 0.72	<0.0001
FT3 (pg/dL) (normal: (230-619 pg/dL)	298.17 ± 84.63	309.92 ± 89.12	0.1771
FT4 (ng/dL) (normal: 0.7-1.9 ng/dL)	1.44 ± 0.37	1.47 ± 0.38	0.4242
SCH (%)	90 (45.05%)	42 (21.0%)	<0.00001

## Discussion

This study showed a significant association of SCH in patients with CKD. Previously, a study carried out by Rhee et al. [7] on 461,607 patients having stage three to five CKD demonstrated that with a 10 mL/min decrease in the GFR than the approximated value, the chances of clinical hypothyroidism were raised to 18%. Another finding of this study concluded a link between decreased GFR and an increase in the TSH levels; this relation was also reported in our study that serum TSH levels were elevated in CKD patients. This was also anchored by a study carried out by Lo et al. in which people who did not have optimally functional kidneys showed a higher prevalence of subclinical and clinical primary hypothyroidism [8].

Our study also concluded that CKD patients were reported to show a significant association with high BMI, as compared to the group that did not have CKD. In concordance with this finding of our study, it was shown that CKD is more frequent in developing countries because the risk factors like obesity [9] are on the rise in these areas.

Literature has thoroughly provided evidence that impaired thyroid function is known to play a major role in the progression of CKD [10-12]. A possible explanation for this correlation is that the thyroid hormone acts on the whole body, including our renal system. Thyroid hormone affects the development and role of the kidney; the kidney also, in turn, affects thyroid hormone as it plays a role in the excretion of iodine, TSH, and thyrotropin-releasing hormone (TRH), in addition to the deiodination of T4 to form T3 [13,14]. Therefore, when the kidney is affected, just like in CKD, these functions are disrupted.

Literature has thoroughly found a frequent association between hypertension and CKD. Approximately 60-90% of CKD patients are known to have hypertension. A possible explanation for this is that CKD is related to the hyperactivity of the renin-angiotensin-aldosterone system (RAAS), which constricts the vessels, increasing systemic vascular resistance and blood pressure [15]. Our study did not prove any significant relation between hypertension and CKD.

Our study also demonstrated that smoking and CKD have a negative correlation. On the contrary, studies in the past stated the exact opposite [16]. The kidney damage seen in smokers is mostly due to hemodynamic and non-hemodynamic changes [17,18]. The non-hemodynamic changes due to smoking that lead to a damaged kidney are oxidative stress, decreased bioavailability of nitric oxide, increased endothelin 1 concentration, tubular cell damage, and increased vasopressin secretion [18]. Smoking also causes insulin resistance [19], which, in turn, decreases the GFR [20], leading to improper functioning of the kidneys with the passage of time. Hemodynamic fluctuations lead to a temporary but considerable rise in blood pressure due to smoking [21,22]. The temporary fluctuations that lead to increased blood pressure lead to advanced kidney diseases [23,24]. To identify the root cause of the discrepancy in our results, frequency, duration of smoking, and lifestyle could be different.

Our study has a few limitations. Since this study was a cross-sectional study, it could not determine the cause-and-effect relationship between SCH and CKD. This study was conducted in a single center; therefore, future studies with a larger sample size and involving multiple centers should be done to confirm the findings of our study. Moreover, follow-up studies should also be carried out to study the long-term effects.

## Conclusions

Among the many complications of CKD, SCH is a major one. Progression of CKD is positively correlated with increased incidence of SCH. Therefore, there is a need to formulate guidelines involving routine screening of CKD patients for SCH. Furthermore, multidisciplinary management, including a team of endocrinologists and nephrologists, is advised to keep a regular check on these patients.
